# Clinical characteristics and multimodal imaging insights of coronary involvement in immunoglobulin G4–related disease

**DOI:** 10.3389/fimmu.2025.1685508

**Published:** 2025-12-04

**Authors:** Guan Wang, Yaqi Du, Yun Bai, Yimo Zhou, Shuang Ding, Xinrui Wang, Yibin Xie, Hsin-jung Yang, Debiao Li, Zhaoyang Fan, Guoguang Fan, Zhe Lou, Jiayi Wei, Yingxian Sun

**Affiliations:** 1 Department of Radiology, The First Hospital of China Medical University, Shenyang, China; 2 Department of Rheumatology and Immunology, The First Hospital of China Medical University, Shenyang, China; 3 Biomedical Imaging Research Institute, Cedars Sinai Medical Center, Los Angeles, CA, United States; 4 Radiology and Radiation Oncology, Keck School of Medicine, University of Southern California, Los Angeles, CA, United States; 5 Department of Echocardiography, The First Hospital of China Medical University, Shenyang, China; 6 Key Laboratory of Cell Biology and Key Laboratory of Medical Cell Biology, Department of Developmental Cell Biology, China Medical University, Shenyang, China; 7 Department of Cardiovascular Medicine, The First Hospital of China Medical University, Shenyang, China

**Keywords:** IgG4-related disease, IgG4-related coronary arteritis, imaging, prognosis, cardiovascular

## Abstract

**Introduction:**

Coronary involvement in immunoglobulin G4–related disease (IgG4-RD) has remained underexplored despite its risk posed in terms of major adverse cardiovascular events (MACEs). The study provides a comprehensive review, particularly focusing on multimodal imaging characteristics and clinical applicability.

**Methods:**

A systematic review was conducted on IgG4-related coronary involvement, supplemented by serial cases from our center included. We analyzed clinical features and multimodal imaging, focusing on the presence or absence of cardiovascular symptoms.

**Results:**

A total of 134 IgG4-RD patients with coronary involvement were included and analyzed, including 118 from the literature and 16 from our center. Seven (5%) patients died from secondary myocardial ischemia/infarction. Coronary anomalies commonly affected the left anterior descending artery (LAD) (79%) and presented as diffuse wall thickening or periarterial soft tissue encasement (85%). Stenosis was frequent (47%) and often secondary. Symptoms, primarily induced by myocardial ischemia or infarction (84%), were largely due to stenosis (68%). Chest computed tomography (CT) and coronary computed tomography angiography (CTA) were the primary imaging modalities (81%), particularly in symptomatic cases (88%). Positron emission tomography-computed tomography (PET-CT) was applied in 55 patients (41%) and often in asymptomatic cases (51%). CMR, though less adopted (23%), demonstrated potential in detecting coronary lesions (77%). Glucocorticoid therapy is the most common (76%), with the best response of periarterial encasement (66%). Surgery was less common (32%), primarily being applied to aneurysms (63%).

**Conclusion:**

Coronary involvement in IgG4-RD presents four phenotypes, sometimes with an insidious onset and as the sole affected site, poses a potential risk for MACEs. Multimodal imaging is essential for early diagnosis and effective monitoring, with coronary CMR showing promise for early detection without the risk of radiation-induced inflammation and fibrosis.

## 1 Introduction

Immunoglobulin G4–related disease (IgG4-RD) is a unique immune-mediated chronic fibro-inflammatory disorder characterized by elevated serum IgG4 levels and lymphoplasmacytic infiltration, predominantly consisting of IgG4-positive plasma cells, within the affected tissues (1). IgG4-RD shows a broad spectrum of organ involvement, with aortitis/peri-aortitis being the most commonly reported in the cardiovascular system (2, 3). However, vascular inflammation is common in IgG4-RD, including in the coronary arteries, which, as medium-sized vessels, have received comparatively limited attention. Notably, the coronary arteries are the second most frequently affected extra-aortic vessels in patients with periarteritis (4). Coronary involvement in IgG4-RD can result in life-threatening complications, including aneurysmal rupture and ischemic heart disease.

Identifying typical sites of involvement is essential for diagnosing IgG4-RD (5). Imaging serves as a crucial tool for the non-invasive detection of involved organs, particularly in areas where biopsy is challenging, such as coronary artery lesions. However, due to an incomplete understanding of this uncommon site and suboptimal imaging assessment, IgG4-related coronary involvement has been underestimated in clinical practice and remains poorly understood. Accordingly, the literature on coronary involvement in IgG4-RD was reviewed, spanning the past 24 years, and supplemented with magnetic resonance (MR) data from a center on IgG4-RD patients with typical coronary involvement. The research aims to elucidate the clinical features and further highlight the imaging characteristics and their clinical applicability.

## 2 Materials and methods

### 2.1 Literature review

#### 2.1.1 Search strategy

The exploration of the literature involved a meticulous search across the PubMed/Medline, Scopus, and Web of Science databases, specifically targeting English-language publications. The MeSH term “Immunoglobulin G4–Related Diseases” along with its related Entry Terms was applied, combined with the MeSH term “Coronary artery” and its corresponding Entry Terms using the Boolean operator “AND”. Additionally, references cited in the papers initially selected to uncover any additional relevant literature for inclusion in the study were reviewed. The outcomes of these search strategies are listed in **Supplementary Table S1**.

#### 2.1.2 Study selection and study screening

**Figure 1:**
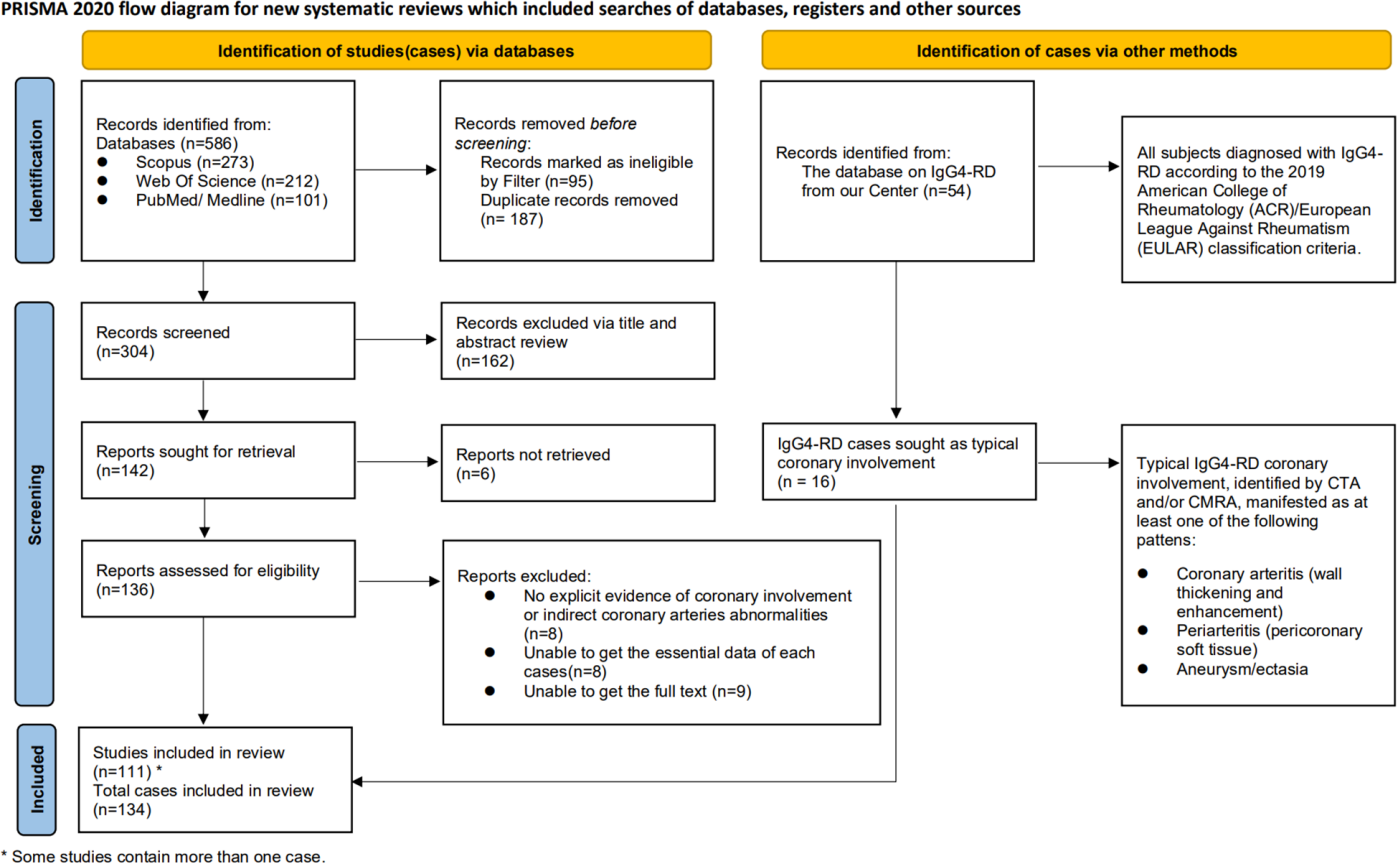
PRISMA diagram for the selection of papers.

The design, data analysis, and interpretation of this study were conducted on the basis of the preferred reporting items for systematic reviews and meta-analyses (PRISMA) guidelines (6) (**Figure 1**). The study eligibility was defined employing the population, intervention, comparator, and outcome framework (7). Detailed inclusion and exclusion criteria are outlined in **Supplementary Table S2**. Publication Date: Articles published between 2000 and 2024.

Two independent reviewers (Du YQ and Bai Y) evaluated the literature abiding by the inclusion criteria. Titles and abstracts of the retrieved articles were screened, and a quality assessment was carried out to identify potentially relevant studies. Each study was assessed for quality and risk of bias using the Joanna Briggs Institute checklist (8).

### 2.2 Retrospective cases review

A series of 77 patients identified as IgG4-RD from a center (database of The First Hospital of China Medical University, 2021-2024) were selected, conforming to the classification criteria for 2019 American College of Rheumatology/European League Against Rheumatism (5), with 21 of them suspected to show coronary involvement after systematic examination based on the patterns described in the previous literature (9). The charts of all patients were reviewed and the IgG4-RD patients with typical coronary involvement were extracted based on radiologic evidence as previously described (10). The case reports were reviewed and approved by the ethics committee of The First Affiliated Hospital of China Medical University. Written informed consent was obtained from all included patients.

### 2.3 Data extraction

The collected medical records include patient demographics (age and sex), clinical characteristics (medical history, treatment details, laboratory and imaging data), assessment of coronary artery lesions, clinical outcomes (response, relapse and remission), and documentation of adverse events.

### 2.4 Statistical analysis

Due to the high publication bias, a highly reliable statistical analysis could not be performed. For illustrative purposes, however, an analysis of a few relevant variables was performed. Normality of distributions was assessed using the Kolmogorov-Smirnov test. Categorical data are presented as percentages, and continuous variables are expressed as mean ± standard deviation or median (interquartile range), as per the data distribution. Comparisons between independent samples were made by virtue of one-way analysis of variance, Kruskal-Wallis test, or chi-squared test with Bonferroni *post-hoc* adjustments and Fisher’s exact tests, as appropriate for the type of data. All tests were two-tailed, with a *p*-value of less than.05 considered statistically significant.

## 3 Results

A total of 586 articles were retrieved, of which 111 met the inclusion criteria and were ultimately included (9, 11–120). These articles reported a total of 118 IgG4-RD patients. Combined with 16 IgG4-RD patients with typical coronary involvement from our center, the total number of cases analyzed reached 134. Detailed information on the patients from the center is provided below and summarized in **Table 1**.

**Table 1:** Characteristics of IgG4-RD patients with coronary artery involvement from the center.

Cases	Age, years	Sex	Comorbidities	Smoking history	Symptoms	Coronary abnormalities	Other cardiovascular involvement	Number of organ involvements	Serum IgG4, mg/dL (before and after treatment)		Serum IgG, mg/dL (before and after treatment)		ESR, mm/h (before and after treatment)		CRP, mg/L (before and after treatment)	
Case 1	63	Male	CAD	+	None	LAD: Type2	Aorta, Pericardium, Myocardium	10	2560	242	3071	1035	50	2	1.0	2.2
Case 2	49	Male	Asthma, Allergic rhinitis	–	Dyspnea, Chest distress	LAD: Type1	None	7	1549	74	1549	948	5	4	1.2	3.0
Case 3	63	Male	Hypertension	–	None	LAD, RCA: Type1	Myocardium, mildly reduced RVEF% and LVEF%	7	415		2079	–	–	–	1	–
Case 4	73	Female	None	–	None	RCA: Type1	Myocardium	4	805	–	–	–	30	–	1.5	–
Case 5	66	Female	None	–	None	LAD: Type1	None	6	634	–	1220	–	28	–	7.6	–
Case 6	68	Female	None	+	None	RCA: Type1	Pulmonary artery	6	1410	354	1991	1298	20	9	0.69	1.48
Case 7	56	Male	None	–	None	RCA: Type1	None	2	1130	405	1419	1105	10	8	2.2	2.0
Case 8	67	Male	None	–	None	LAD: Type1, 4	None	7	3390	–	2464	–	36	–	3.7	–
Case 9	68	Male	None	–	Palpitation, Shortness of breath	LAD, LCX, RCA: Type1	Myocardium, Reduced RVEF% and LVEF%	5	1840	347	1977	1080	12	2	2.4	1.3
Case 10	58	Male	None	–	Palpitation, Shortness of breath, Chest distress	RCA: Type1,4; LAD: Type1	None	9	7030	799	5648	856	>90	15	7.2	3.1
Case 11	66	Male	None	–	None	LAD: Type1	None	6	1360	–	2431	–	62	–	6.2	–
Case 12	60	Male	None	–	None	LAD: Type1	None	9	4030	–	3140	–	50	–	3.5	–
Case 13	71	Female	None	–	None	LAD: Type1	None	4	3020	–	2753	–	77	–	4.7	–
Case 14	69	Male	None	–	None	LAD, RCA: Type1	Pericardium	6	1940	–	1861	–	18	–	3.2	–
Case 15	52	Male	None	–	None	LCX: Type1	None	5	1300	–	1593	–	12	–	3.4	–
Case 16	62	Male	None	–	None	LAD: Type1	Reduced RVEF%	7	2030	–	2196	–	28	–	2.7	–

Type 1: wall thickening, Type 2: periarterial soft tissue encasement, Type 3: aneurysm/ectasia and Type 4: stenosis.

LAD: left anterior descending artery; LCX: left circumflex artery; RCA: right coronary artery; CAD: coronary artery disease; LVEF: left ventricle ejection fraction; RVEF: right ventricle ejection fraction.

For further analysis, all included patients were classified into two groups based on their clinical presentation of coronary involvement: the symptomatic group (*n* = 75) and the asymptomatic group (*n* = 59) (**Table 2**).

**Table 2:** Characteristics of IgG4-RD with coronary involvement.

Characteristics	Symptomatic (*n* = 75)	Asymptomatic (*n* = 59)	*p*-value
Age, years	63 [54-70]	63 [58-71]	.23
Sex (male)	63 [84]	49 [83]	.88
Disease duration, years	5 [1-10]	5 [2-8]	.45
Hypertension	12 [16]	13 [22]	.37
Diabetes	6 [8]	8 [14]	.30
Atherosclerosis	28[37]	31[53]	.08
Number of organs involvement	3 [2-4]	5 [3-6]	**<.001**
Asthma	6[8]	8[14]	.30
Myocardial ischemia/infarction	63 [84]	7 [12]	**<.001**
Large vessel involvement			
Aorta	23 [31]	23 [39]	.31
Others	15 [20]	13 [22]	.77
Coronary involvement			
LAD	58 [77]	48 [81]	.57
LCX	37 [49]	27 [46]	.68
RCA	60 [80]	35 [59]	**.009**
Coronary abnormalities			
Type 1	30 [40]	28 [48]	.39
Type 2	36 [48]	26 [44]	.65
Type 3	43 [57]	23 [39]	**.04**
Type 4	45 [60]	18 [31]	**<.001**
Peak IgG4, mg/dL	799 [278-1761]	1410 [785-2600]	**.006**
Peak IgG, mg/dL	2080 [1618-4027]	2464 [1991-3864]	.39
Peak IgE, IU/mL	1014 [338-1709]	743 [361-2405]	.76
ESR, mm/h	58 [16-95]	52 [22-80]	.71
CRP, mg/L	8.9 [4.1-24.8]	3.6 [1.6-9.2]	**.01**
Imaging examination			
CT/CTA	66 [88]	42 [71]	**.02**
ICA	41 [55]	16 [27]	**.001**
CMR	13 [17]	18 [31]	.07
PET-CT	25 [33]	30 [51]	**.04**
IVUS	12 [16]	5 [9]	.19
Echocardiography	20 [27]	24 [41]	.09

Values are *n* [%], or median [interquartile range].

One-way analysis of variance and Kruskal-Wallis with *post-hoc* tests for differences.

*p*-value for the one-way analysis of the whole cohort.

Type 1: wall thickening, Type 2: periarterial soft tissue encasement, Type 3: aneurysm/ectasia and Type 4: stenosis. Statistically significant values are shown in bold.

LAD: left anterior descending artery; LCX: left circumflex artery; RCA: right coronary artery; ESR: erythrocyte sedimentation rate; CRP: C-reactive protein; CT: computed tomography; ICA: invasive coronary angiography; CMR: cardiac magnetic resonance; PET-CT: positron emission tomography-computed tomography; IVUS: intravascular ultrasound.

### 3.1 Characteristics of the patients included in the analysis

The patients were predominantly middle-aged males, consistent with the demographic profile of IgG4-RD patients reported in the literature (121). Notably, three younger patients (aged 13, 15, and 22 years) in the cohort exhibited significant coronary involvement (33, 80, 85), with two experiencing secondary myocardial infarction and reduced cardiac function (80, 85). There were no significant differences in age distribution across groups. The time from disease onset to the detection of coronary abnormalities varied widely, ranging from 12 weeks to 25 years.

Most patients (85%) presented multi-organ involvement in addition to coronary lesions, with lymph nodes being the most frequently affected site (27%). Within the cardiovascular system, the aorta was most commonly affected (34%). Detailed information on extra-cardiovascular involvement is provided in **Supplementary Table S3**. Interestingly, patients in the asymptomatic group had a significantly larger number of organs involved compared to those in the symptomatic group (*p* <.001).

### 3.2 Diagnosis of coronary artery involvement

Among the 134 cases studied, 20 patients (15%) presented with isolated coronary involvement, while the remaining patients had already been diagnosed with IgG4-RD through the identification of involvement in other organs before coronary lesions were detected. The diagnosis of IgG4-RD coronary artery involvement primarily relied on cardiovascular-related clinical symptoms, characteristic imaging findings, and elevated serum IgG4 levels, with biopsy occasionally performed for further confirmation.

In the cohort, 91 patients received a definitive IgG4-RD diagnosis confirmed by biopsy, with coronary involvement pathologically validated in 38 cases (42%), including 12 with isolated coronary lesions. Similar to other organs affected by IgG4-RD, the histologic hallmark of coronary involvement included diffuse lymphoplasmacytic infiltrates with abundant IgG4-positive plasma cells, a high IgG4-to-IgG plasma cell ratio, and storiform fibrosis.

While the 16 cases of IgG4-RD with coronary involvement from the center were not biopsy-confirmed, they exhibited no evidence of coronary artery diseases (CADs) and consistently displayed characteristic imaging features of IgG4-RD coronary involvement. Furthermore, the majority of these patients showed regression of coronary lesions following disease-modifying therapies, further supporting the diagnosis.

### 3.3 Imaging modalities for evaluating coronary artery involvement in IgG4-RD

Chest computed tomography (CT) and coronary computed tomography angiography (CTA) were the most commonly used imaging modalities, identifying coronary abnormalities in 108 patients (81%). Notably, 42 of these patients (39%) were incidentally diagnosed in the absence of symptoms (**Table 2**). Invasive coronary angiography (ICA) was the second most frequently utilized imaging technique, performed in 57 patients (43%), primarily as a complementary method to CT for confirming luminal stenosis or ectasia and assessing its severity. Positron emission tomography-computed tomography (PET-CT) was applied in 55 patients (41%), while intravascular ultrasound (IVUS) was performed in only 17 patients (13%). In the symptomatic group, CT/CTA and ICA were more frequently adopted compared to the asymptomatic group (CT/CTA: 88% *v*. 71%, *p* = .005; ICA: 55% *v*. 27%, *p* = .001). Conversely, PET-CT was more frequently employed in the asymptomatic group compared to the symptomatic group (51% *v*. 33%, *p* = .04).

Cardiovascular magnetic resonance (CMR) was not routinely used in clinical practice for evaluating coronary involvement, with only 31 patients (23%) assessed in this cohort. Among the 15 cases identified in the literature, coronary lesions were detected in eight cases through CMR. However, all 16 patients from the center underwent CMR, with coronary lesions consistently identified via contrast-enhanced coronary MR angiography (MRA).

Follow-up imaging was conducted on 57 post-treatment patients. Of these, 45 patients (79%) underwent chest CT/CTA to assess changes in coronary lesion size and luminal alterations. PET-CT was applied in 19 patients (33%) to evaluate inflammatory activity through changes in 18F-FDG uptake. At the center, six patients were followed up with CMR, allowing for the evaluation of coronary lesion size and the extent of coronary wall enhancement.

#### 3.3.1 Characteristics of coronary involvement in IgG4-RD

**Figure 2:**
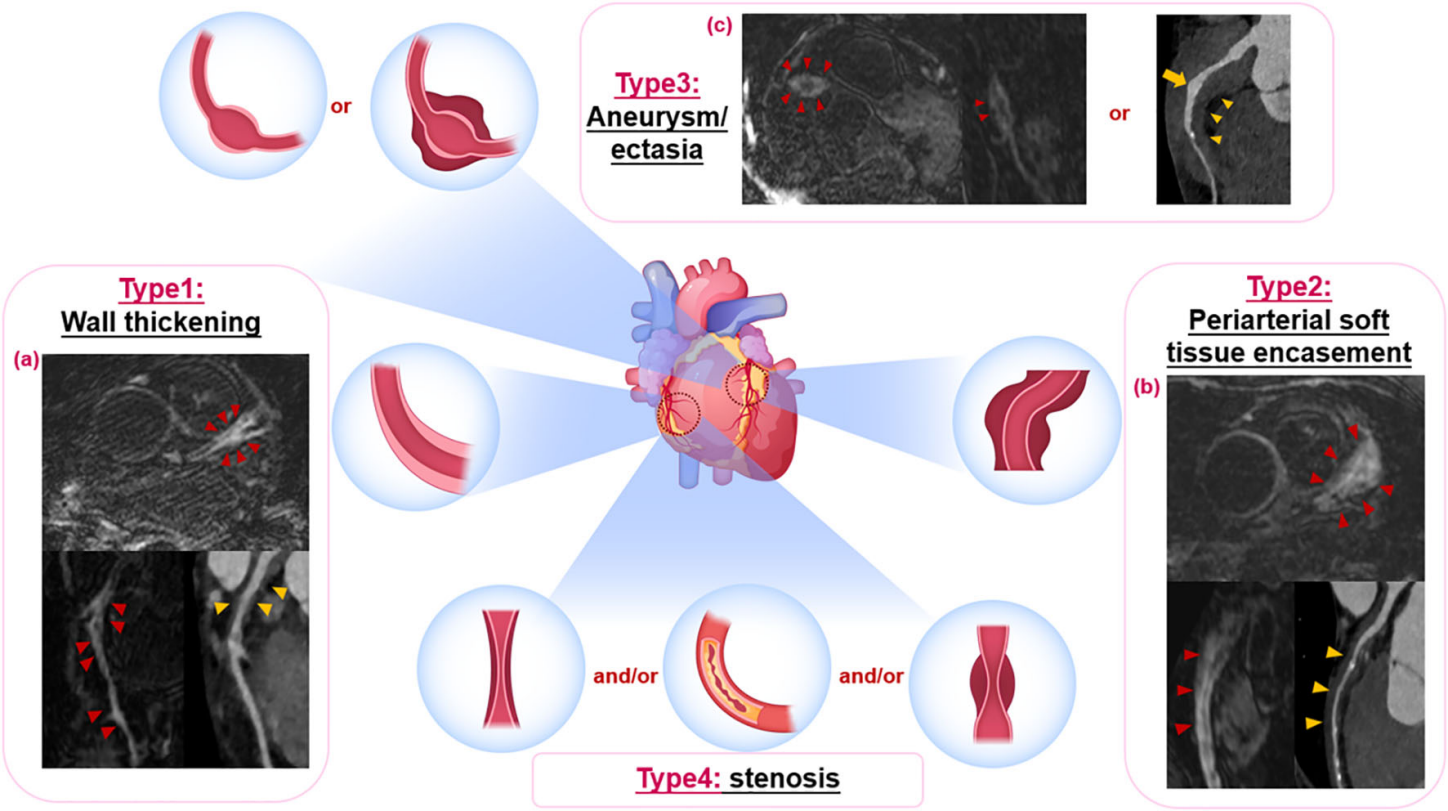
Four types of IgG4-related coronary artery lesions by multimodal imaging evaluation. Type 1: wall thickening, Type 2: periarterial soft tissue encasement and Type 3: aneurysm/ectasia may coexist; Type 4: stenosis generally secondary to other types or atherosclerosis. **(a)** Thickening of the LAD coronary wall (red arrowheads on CMR and yellow arrowheads on CTA), with contrast enhancement on coronary CMR angiography even in segments without apparent wall thickening on CTA (red arrowheads), accompanied by secondary luminal stenosis. **(b)** Periarterial soft tissue encasement of LAD, resembling the “Pigs-in-a-Blanket Coronary Artery” on CTA (yellow arrowheads), coexists with wall thickening and contrast enhancement on coronary CMR angiography (red arrowheads), accompanied by secondary luminal stenosis. **(c)** Aneurysm formation of RCA with wall thickening and contrast enhancement on coronary CMR angiography (red arrowheads); localized ectasia of LAD (yellow arrow) with periarterial soft tissue formation (yellow arrowheads). LAD: left anterior descending artery; RCA: Right coronary artery; CMR: cardiac magnetic resonance.

Consistent with the descriptions of IgG4-related coronary involvement by Akiyama et al. and Katz et al. (10, 122), four types of coronary artery involvement were identified through multimodal imaging, often with multiple types coexisting (**Figure 2**):

Type 1: Wall thickening (43%) – Characterized by coronary wall thickening with contrast enhancement on CT/MRI (arteritis). Type 2: Periarterial soft tissue encasement (46%) – Appears as localized or diffuse peri-coronary soft tissue thickening (periarteritis), sometimes described as pseudo-tumors, which may cause luminal narrowing. Type 3: Aneurysm/ectasia (49%) – Characterized by localized or continuous luminal dilation, typically accompanied by mild coronary wall thickening or periarterial soft tissue encasement. Larger dilations are prone to mural thrombus formation (35%), potentially causing luminal occlusion due to thrombus detachment. Type 4: Stenosis (47%) – Generally considered a secondary phenomenon, often following Type 2 lesions.

These three types (1-3) frequently exhibited elevated uptake on 18F-FDG PET/CT and contrast enhancement on CT/CMR. Additionally, 44% of cases had concomitant atherosclerosis, with stenosis in some cases directly resulting from localized atherosclerosis (19%).

In comparing the two groups, the incidence of aneurysm/ectasia (Type 3) and stenosis (Type 4) was significantly higher in the symptomatic group than the asymptomatic group (symptomatic *v*. asymptomatic group: Type 3: 57% *v*. 39%, *p* = .04; Type 4: 60% *v*. 31%, *p* <.001). Additionally, the most frequently affected site of coronary involvement across the cohort was the left anterior descending artery (LAD) (79%), but the symptomatic group demonstrated a higher incidence of right coronary artery (RCA) involvement (symptomatic *v*. asymptomatic group: 80% *v*. 59%, *p* = .009). This may be attributed to the fact that involvement of multiple coronary arteries is more likely to induce cardiovascular symptoms, while multiple types of coronary lesions are more commonly overlapped in the LAD.

#### 3.3.2 CMR manifestations of cardiovascular involvement and coronary MRA techniques

A total of 31 patients underwent CMR examination, with coronary involvement identified in 24 cases (77%), including eight cases from the literature review (**Supplementary Table S4**). CMR evaluation revealed three primary types of coronary involvement: wall thickening (75%), periarterial soft tissue encasement (29%), and aneurysm/ectasia (25%), with frequent overlap between these patterns; however, without the use of coronary MRA, a technique not routinely employed in IgG4-RD imaging evaluation, identifying coronary arterial luminal stenosis based solely on routine CMR remained challenging. Among the reviewed cases in the literature, only one case (**Supplementary Table S4**, case 7) underwent coronary MRA examination. All patients at the center received contrast-enhanced coronary wall MR imaging (123), which facilitated the detection of coronary wall abnormalities, including mild wall thickening. The most characteristic CMR findings in this cohort included enhancement of thickened coronary walls, peri-coronary masses, and aneurysm walls. Multi-parameter MR imaging also played a critical part in the identification of intracavitary thrombosis (**Supplementary Table S4**, cases 5 and 6).

**Figure 3:**
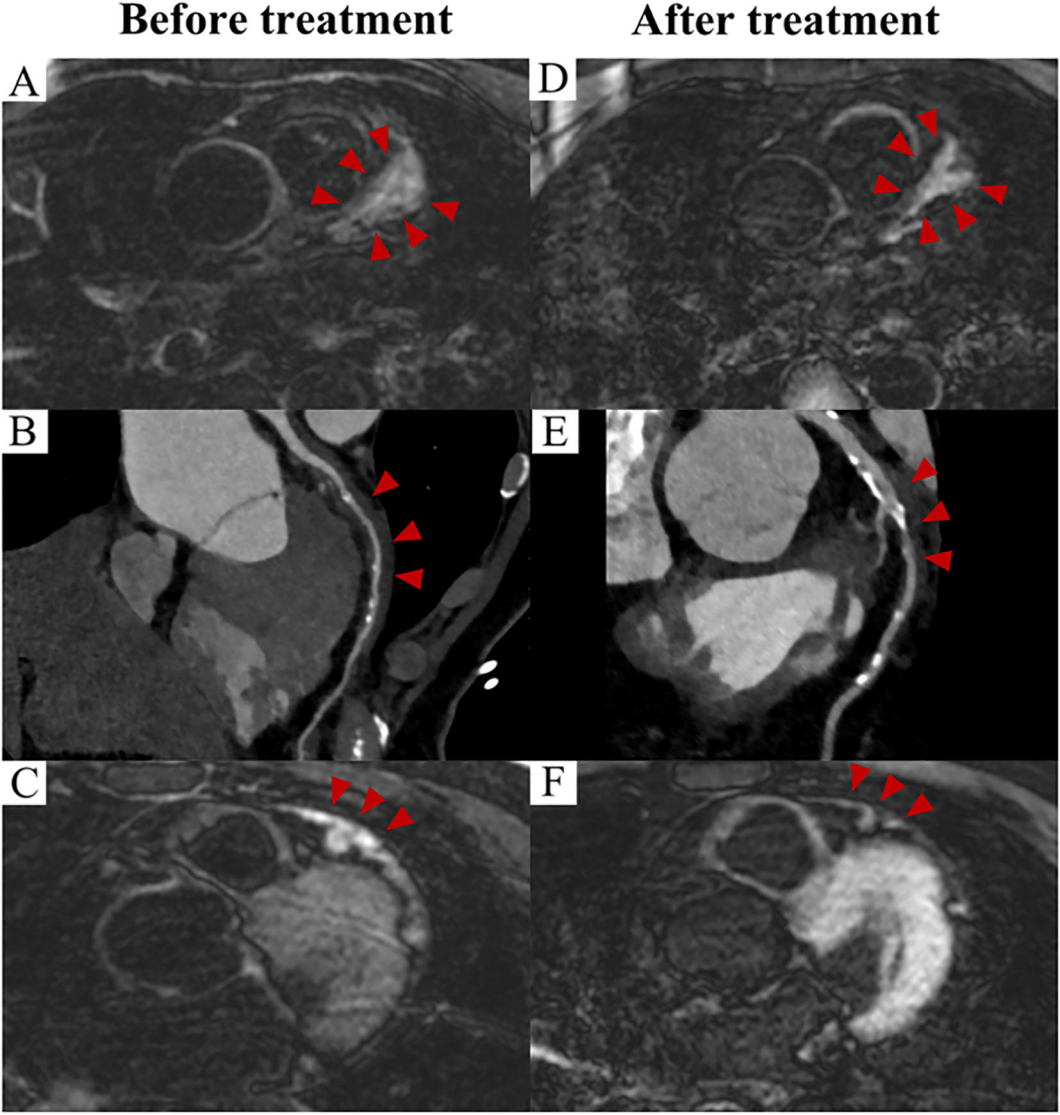
Prognosis of IgG4-related coronary involvement evaluated by imaging (Case 1). CMR image demonstrate wall thickening and enhancement of the LAD before treatment **(A)**, and a reduction in both wall thickening and enhancement were observed after two years of treatment **(D)**. (red arrowheads) CTA images demonstrate the corresponding findings **(B, E)**. (red arrowheads) Pericardial thickening with enhancement was evident on CMR before treatment **(C)**, with no abnormality observed after treatment **(F)**. (red arrowheads). CMR: cardiac magnetic resonance; CTA: computed tomography angiography; LAD: left anterior descending artery.

Apart from coronary involvement, CMR identified both inflammatory non-ischemic myocardial abnormalities (19%) and secondary ischemic myocardial abnormalities (13%), even among asymptomatic patients. Non-ischemic myocardial abnormalities were typically characterized by subepicardial or mid-myocardial late gadolinium enhancements (LGEs) and/or elevated T2 values, which were often directly associated with the inflammatory infiltration of the disease rather than secondary to the coronary involvement. Ischemic myocardial abnormalities, on the other hand, shown as subendocardial or transmural LGEs, with coronary CTA/ICA confirming the culprit coronary arteries in three patients. Furthermore, CMR detected pericardial involvement in one patient from the center, who showed significant remission following treatment (**Figures 3C, F**).

### 3.4 Complications of coronary involvement

Chest tightness and chest pain/angina were the most common clinical symptoms. Patients with myocardial ischemia/infarction were significantly more common in the symptomatic group than the asymptomatic group (84% *v*. 12%, *p* <.001), with this condition most frequently observed in symptomatic patients with Type 4 involvement (68%). This indicates that myocardial ischemia/infarction caused by coronary stenosis may represent the primary cause of symptoms in these patients. In the asymptomatic group, four patients experienced sudden death due to myocardial ischemia/infarction (three with Type 4 involvement and one with Type 3), three of whom had a documented history of prior myocardial infarction.

### 3.5 Laboratory findings

In a cohort of 112 patients with known serum IgG4 levels, the majority showed markedly elevated serum IgG4 (95%), with a median level of 1160 mg/dL. Interestingly, it seems that the severity of clinical manifestations did not appear to correlate with the degree of elevation in serum IgG4 levels: the serum IgG4 level in the asymptomatic group was higher than that of the symptomatic group (1410 mg/dL *v*. 799 mg/dL, *p* = .006). Notably, six individuals (6%) with normal serum IgG4 levels still presented significant symptoms of coronary artery involvement. Serum C-reactive protein (CRP) levels were recorded in 48 cases across the cohort (median: 4.9 mg/L), with 25 patients (52%) exhibiting values above the normal range. The serum CRP level in the symptomatic group was higher than that of the asymptomatic group (8.9 mg/L *v*. 3.6 mg/L, *p* = .01). Other laboratory results were recorded in less than half of the cases, with over 70% within the normal range, revealing no significant differences between the two groups (**Table 2**).

### 3.6 Treatment and prognosis

The majority of IgG4-RD patients with coronary artery involvement were treated with glucocorticoids (76%), with more than 50% demonstrating remission of coronary lesions. The use of immunosuppressants alone (21%) or rituximab alone (7%) was uncommon, as these therapies were typically combined with glucocorticoids.

Surgical treatment is generally recommended for giant aneurysms (> 20 mm) or multiple aneurysms (124). Among patients with known lesion sizes, 28 (42%) had aneurysms larger than 20 mm, with 16 undergoing surgery, and most achieved remission or stabilization of their lesions (69%). Across the entire cohort, however, surgical treatment for coronary lesions was less frequent (32%), possibly because of operability limitations. Surgical cases included aneurysms (37%), periarterial soft tissue encasement (23%), a combination of both (26%), and wall thickening (14%). Among these, five patients (Type 3 combined with Type 1/2/4) received surgical treatment after failing to respond to glucocorticoid therapy.

The most commonly employed surgical approach was lesion excision combined with coronary artery bypass grafting (33%), with most patients receiving routine postoperative glucocorticoid therapy. The prognosis for surgical treatment was favorable, with 65% of patients achieving relief or stabilization of their condition. However, four cases (9%), including three with Type 3 and one with Type 2, demonstrated enlarged coronary lesions after coronary artery bypass grafting. Notably, two of these patients had not received glucocorticoid therapy (56, 58, 60, 92, 97).

Overall, most patients had a favorable prognosis after medical and/or surgical treatment, evidenced by reductions in peri-coronary mass size, aneurysm diameter, or alleviation of wall thickening on CT/CTA/CMR imaging (**Figure 3**), as well as reduced FDG uptake on 18F-FDG PET/CT scans. Prognosis varied across different types of coronary artery involvement, as displayed in **Table 3**. Type 2 involvement demonstrated the best prognosis, with the highest improvement rate (66%) and the lowest rate of progression (8%). Type 3 had a relatively higher rate of progression (15%).

**Table 3:** Prognosis of IgG4-RD with different coronary abnormalities.

Type of coronary involvement	Number of patients	improved	Progressive	Stable	Dead	Unknown
Type 1	58	19[33]	5[9]	11[19]	7[12]	16
Type 2	62	41[66]	5[8]	7[11]	5[8]	4
Type 3	66	33[50]	9[14]	11[17]	3[5]	10
Type 4	63	34[54]	5[8]	9[8]	8[13]	7

Type 1: wall thickening, Type 2: periarterial soft tissue encasement, Type 3: aneurysm/ectasia and Type 4: stenosis.

### 3.7 Characteristics of death cases

The total number of deceased patients in the cohort was 13 (10%), of whom 7 (54%) died from cardiac causes, all directly attributed to myocardial ischemia/infarction. The detailed characteristics are listed in **Table 4**. Among these, three deaths were due to wall thickening or periarterial soft tissue encasement, two were induced by thrombosis formation associated with atherosclerosis, and the remaining two resulted from thrombus formation within coronary artery aneurysms, leading to severe narrowing or occlusion of the coronary artery lumen. Notably, five patients exhibited no discernible signs of IgG4-RD involvement beyond the coronary arteries before death; postmortem examinations confirmed IgG4-RD influencing the coronary arteries. Additionally, four patients had not experienced any cardiovascular-related symptoms before their sudden death.

**Table 4:** Characteristics of dead patients.

Study (reference)	Age at the discovery of CAI	Sex	Type of coronary artery involvement	Coronary involvement	Peak IgG4, mg/dL	Peak IgG, mg/dL	Peak IgE, IU/mL	ESR, mm/h	CRP, mg/L	Treatment	Cause of death	Other organs involvement	Imaging examination
Ramdin et al. (11)	84	Male	Type 1	LAD, LCX, and RCA	1160 mg/dL	2890 mg/dL			Normal	Glucocorticoid and rituximab	Aspiration pneumonia	Kidney, ureter, biliary tract, pancreas, retroperitoneum, retroorbital tissue, lymph node	CT
Inokuchi et al. (12)	38	Male	Type 1 and type 4	LCX and RCA	109 mg/dL	1563 mg/dL			3.0 mg/L	NA	Coronary thrombosis led to ischemic heart disease	NA	CT
Patel et al. (13)	53	Male	Type 1, type 2 and type 4	LAD, LCX, and RCA	NA					NA	Coronary thrombosis led to myocardial infarction	NA	NA
Paratz et al. (18)	48	Male	Type 1 and type 4	Multi-vessel	NA					NA	Coronary stenosis led to ischemic heart disease	Spleen enlargement, retroperitoneal soft tissue adjacent to the aorta	NA
Paratz et al. (18)	50	Male	Type 2 and type 4	LAD	NA					NA	Coronary stenosis led to myocardial ischemia	NA	NA
Gutierrez et al. (19)	54	Male	Type 3	LAD and RCA	NA					NA	Thrombosis in aneurysm led to myocardial infarction	NA	NA
Bukiri et al. (20)	55	Male	Type 1 and type 4	LAD and LM	NA					Glucocorticoid	Coronary occlusion led to myocardial ischemia	NA	NA
Chan et al. (67)	73	Male	Type 3	LAD, LM and RCA	NA					Glucocorticoid	Thrombosis in aneurysm led to myocardial ischemia	Surrounding soft tissue mass of right internal iliac artery	CT
Yardimci et al. (9)	61	Male	Type 1 and type 4	RCA	148 mg/dL				15.3 mg/L	Glucocorticoid	Heart failure, sepsis, systemic multi-organ failure	Orbital inflammation	CT and echocardiography
Urabe et al. (38)	84	Male	Type 2 and type 3	LAD, LCX, and RCA	2630 mg/dL	8194 mg/dL				Glucocorticoid	Rupture of thoracic aortic aneurysm	NA	CT, DSA, IVUS and PET
Tajima et al. (52)	68	Male	Type 2	LAD, LCX, and RCA	2390 mg/dL	4305 mg/dL	9716 IU/mL	97 mm/h	9.7 mg/L	Glucocorticoid	Rupture of splenic aneurysm	Multiple systemic aneurysms of right internal carotid artery, bilateral subclavian arteries, left axillary artery, left brachial artery, common hepatic artery, and splenic artery; Salivary glands involvement	CT and PET-CT
Ibrahim et al. (100)	70	Male	Type 2 and type 4	LAD, LCX, and RCA	3030 mg/dL					Glucocorticoid and rituximab	Toxic megacolon	Orbital inflammatory pseudo-tumor, abdominal aortic aneurysm, and bilateral common iliac artery involvement	CT, PET-CT and echocardiography
Fujii et al. (102)	77	Male	Type 1 and type 4	LCX	1750 mg/dL	4813 mg/dl				Glucocorticoid	Shock and metabolic acidosis	Multi-organ involvement includes submandibular gland, sigmoid mesentery, retroperitoneal soft tissue, abdominal aorta, liver, extrahepatic bile duct, bilateral lungs, bilateral kidneys, bladder, prostate, epicardium, and lymph nodes	CT

Type 1: wall thickening, Type 2: periarterial soft tissue encasement, Type 3: aneurysm/ectasia and Type 4: stenosis.

LAD: left anterior descending artery; LCX: left circumflex artery; RCA: Right coronary artery; ESR: erythrocyte sedimentation rate; CRP: C-reactive protein; CT: computed tomography; DSA: digital subtraction angiography; PET-CT: positron emission tomography-computed tomography; IVUS: intravascular ultrasound.

## 4 Discussion

In this review, a detailed analysis of the characteristics of IgG4-RD coronary involvement was provided on the basis of published reports and data from the center.

### 4.1 Epidemiology of IgG4-RD coronary involvement

Clinically, 60%-90% of IgG4-RD cases present with multi-organ involvement, with almost all organs potentially affected. Approximately 10%-30% of cases are complicated by vascular involvement, including arteritis and periarteritis (125), while coronary involvement accounts for only 7% of systemic organ involvement and 10% of vasculitis (126, 127). Despite increasing recognition, the understanding of IgG4-RD coronary involvement as a distinct clinical entity remains limited, and most coronary artery lesions are currently identified through CTA, which is primarily sensitive in detecting Type 2 and Type 3 lesions. However, the insensitivity of conventional imaging techniques to coronary wall inflammation probably results in a significant underestimation of the true extent of coronary involvement in IgG4-RD. In our previous single-center study, coronary MR imaging was utilized for visualizing and quantifying both luminal and wall changes in IgG4-RD coronary vasculitis. About 77% of patients were found to exhibit abnormal coronary wall enhancement, predominantly in a diffuse pattern, suggesting inflammatory involvement (128, 129). This finding indicates that coronary involvement may be more prevalent than currently appreciated and that its true burden remains underestimated.

### 4.2 Mechanisms of IgG4-RD vascular involvement

To date, the pathogenesis of IgG4-RD remains unclear. In recent years, most perspectives have suggested that its occurrence is related to interactions between innate and adaptive immune responses as well as the cooperation between B cells and T cells and the involvement of T follicular helper (Tfh) cells. These interactions can lead to IgG4 class switching, germinal center formation, and plasmablast differentiation, thereby initiating tissue damage and/or fibrosis (130). The typical pathological features of IgG4-RD involving the coronary arteries include coronary wall inflammation and lymphoplasmacytic infiltration (72), resulting in fibrosis and thickening of the coronary wall. Kasashima et al. conducted a retrospective study through the use of a novel histopathological method for whole-slide analysis of IgG4-related abdominal aortic aneurysms (IgG4-AAA). The results indicated that IgG4-AAA should be positioned as adventitial vasculitis with predominant T follicular regulatory and Tfh2 cells, accompanied by the abnormal appearance of arterial tertiary lymphoid organs (ATLOs) (131, 132). ATLOs typically formed in response to chronic inflammation due to infection or autoimmunity (133), and are rarely presented in the adventitia of normal arteries, making their presence a strong indicator of vascular disease (131). The development of ATLOs may sustain inflammatory processes within the arterial walls, driving vascular remodeling, damage, and fibrosis (134). Beyond aortic diseases, ATLOs have also been observed in the adventitia of abnormal medium-sized arteries, including coronary arteries affected by atherosclerosis (135). These findings may provide valuable insights into the pathological mechanisms underlying coronary involvement in IgG4-RD.

### 4.3 IgG4-related coronary involvement *v*. aortic involvement

Coronary involvement and aortic involvement refer to the vessel involvement in IgG4-RD, and have similar manifestations with wall thickening in arteritis, perivascular mass formation in periarteritis. However, they are not identical, especially in cases of periarteritis.

According to clinicopathological patterns, IgG4-RD can be divided into two subtypes: proliferative and fibrotic. These types differ significantly in clinical characteristics and response to glucocorticoid therapy. Lesions in the mediastinum, retroperitoneum and periaortic tissues are commonly associated with the fibrotic subtype (136), while coronary arteritis and periarteritis are more frequently observed in the proliferative subtype (10, 136).

Large vessel involvement in IgG4-RD is suggested as best being classified as primary manifestations of wall thickening (arteritis), and secondary manifestations of perivascular mass formation (periarteritis, mainly idiopathic retroperitoneal fibrosis) (137–139). Recent evidence indicates that idiopathic retroperitoneal fibrosis represents a manifestation of autoimmune or fibro-inflammatory diseases (139, 140). This helps explain why periarteritis/retroperitoneal fibrosis was classified as a secondary type. IgG4-related retroperitoneal fibrosis arises from an autoimmune response, resulting in the accumulation of inflammatory cells, such as lymphocytes and macrophages, in the retroperitoneal region, which release inflammatory and profibrotic mediators that drive fibrous tissue proliferation (136). In patients with periarteritis/retroperitoneal fibrosis, the vascular wall is rarely involved, however, in cases with peri-coronary soft tissue masses, distinct supplying vessel from the coronary artery was observed (58), suggesting that the mass is not entirely isolated from the coronary artery. Furthermore, biopsies have discovered positive cell infiltration in the adventitia of coronary arteries, accompanied by peri-coronary mass formation (38), as the adventitia is the initial site of various vascular diseases, including periarteritis and atherosclerosis (141). IgG4-related coronary arteritis/periarteritis may represent the same entity, both originating from the adventitia, coronary wall thickening may progress to peri-coronary masses as the disease advances. Definitive answers remain elusive due to a lack of longitudinal direct evidence, underlining the need for longitudinal studies integrating clinical and pathophysiological data.

CMR and 18F-FDG PET/CT have been suggested as tools to help distinguish arteritis from periarteritis to some extent (137). However, coronary periarteritis may occasionally present in a diffuse pattern, making it difficult to differentiate from arteritis characterized by diffuse and significant coronary wall thickening. Regardless of this distinction, both conditions increase the risk of coronary stenosis, potentially leading to myocardial infarction and other severe cardiovascular events. Early detection is therefore crucial.

### 4.4 Identification of IgG4-related coronary involvement

Symptomatic IgG4-related coronary involvement frequently manifests as myocardial ischemia or infarction, although the presence of symptoms does not necessarily correlate with number of organs involved. For some patients, coronary involvement may represent the initial and sole manifestation, carrying the risk of severe outcomes or even sudden death. The interval from symptom onset to detection of coronary artery lesions in IgG4-RD varies widely among individuals with different clinical presentations, underscoring the insidious and often overlooked nature of coronary involvement. Early recognition and timely intervention are therefore crucial for managing IgG4-RD-related coronary involvement.

The pathological diagnostic criteria for IgG4-related aortitis/periaortitis include: (1) Histology consistent with aortitis or periaortitis that cannot be readily explained by other processes such as atherosclerosis; (2) At least 50% of plasma cells staining positive for IgG4; and (3) A minimum of 50 IgG4+ plasma cells per 400× high-power field, assessed across at least three fields (142). In most clinical scenarios, pathologically confirming coronary artery involvement in IgG4-RD is particularly challenging due to the difficulty of obtaining biopsy samples from the affected coronary arteries.

#### 4.4.1 Laboratory indicators

Although elevated serum IgG4 levels can support the diagnosis of IgG4-RD, Wallace et al. (143) reported that only 51% of biopsy-confirmed active IgG4-RD cases shad elevated serum IgG4. In the cohort, serum IgG4 levels did not correlate with the presence or absence of symptoms. This highlights the need for early screening to rule out coronary involvement, even in patients with modestly elevated or normal IgG4 levels.

CRP levels were normal in half of the patients, but CRP was associated with clinical symptoms. Most studies noted that patients with IgG4-related coronary arteritis were more likely to exhibit elevated serum CRP compared to those with non-vasculitis IgG4-RD or other autoimmune arteritis (122). However, previous studies did not demonstrate a consistent correlation among CRP levels CRP levels, disease activity, and coronary involvement, a discrepancy that maybe attributable to treatment received by some patients (128).

#### 4.4.2 Diagnostic value of imaging examination

Due to the lack of specific clinical symptoms and laboratory markers, detecting coronary involvement in IgG4-RD is challenging and depends largely on imaging evidence. Multimodal imaging plays a critical role in diagnosing and assessing the prognosis of IgG4-RD coronary involvement. IgG4-RD predominantly affects middle-aged and elderly males, a population already at high risk for coronary atherosclerosis, highlighting the need to differentiate IgG4-RD coronary arteritis from coronary atherosclerosis (144).

##### 4.4.2.1 Coronary CTA and ICA

Coronary CTA is effective in identifying coronary stenosis, tumor-like lesions, coronary ectasia, and aneurysmal formation, and can sometimes assist in differentiating arteritis from atherosclerosis (145). Atherosclerosis on coronary CTA typically manifests as focal plaques within the coronary arteries, leading to luminal narrowing, whereas IgG4-RD coronary arteritis often presents as diffuse, circumferential coronary wall thickening (80). However, the positive coronary CTA findings associated with IgG4-RD coronary arteritis often reflect advanced stages of inflammation, while false negatives in the early stages may cause an underestimation of the true extent and progression of the disease. Notably, coronary atherosclerosis and arteritis can coexist, with arteritis potentially accelerating the progression of atherosclerosis. While ICA can accurately depict coronary luminal anomalies, its ability to assess aneurysms with mural thrombosis is less precise compared to coronary CTA (87). Moreover, ICA is limited in visualizing changes in the coronary artery wall. Despite these constraints, coronary CTA and ICA remain the most commonly used imaging modalities for evaluating symptomatic patients in clinical practice.

##### 4.4.2.2 PET/CT

As IgG4-RD often involves multiple organs, the application of (18)F-FDG PET/CT is not uncommon in clinical practice (18).F-FDG PET/CT could quantitatively evaluate disease activity in CADs, as elevated (18)F-FDG uptake indicates active inflammation. Even if coronary artery masses do not diminish in size after treatment, a reduction in (18)F-FDG uptake may still reflect the effectiveness of treatment (146). Additionally, the immunopathological characteristics of the arterial wall of IgG4-AAA patients suggest that the concentration of FDG uptake observed by PET/CT might reflect the amount or activity of ATLOs (131). Moreover, many asymptomatic patients with coronary involvement were incidentally identified through (18)F-FDG PET/CT, highlighting its value for screening multi-organ involvement. While (18)F-FDG PET/CT is effective in diagnosing large-vessel vasculitis, its diagnostic capacity remains limited to some extent for small and medium-sized vasculitis, including coronary arteritis, due to resolution constraints (147). Thus, combining (18)F-FDG PET/CT with CTA, particularly ECG-gating CTA, may provide a more comprehensive and complementary approach to assessing inflammatory IgG4-related cardiovascular diseases (137).

##### 4.4.2.3 CMR

Although its use in clinical practice remains relatively uncommon, the sensitivity of CMR for early detection of coronary arteritis is increasingly recognized. As a non-invasive imaging modality free from radiation exposure, CMR provides a comprehensive, “one-stop” assessment of the cardiovascular system. This is particularly significant given that radiation exposure may contribute to the progression of IgG4-RD (148). Radiation-induced injury triggers inflammation, ultimately promoting the transdifferentiation of fibroblasts into myofibroblasts (149), a hallmark of fibrosis-related diseases and a key pathogenetic feature of IgG4-RD (150).

Recently, advancements in imaging technology have highlighted promising applications of coronary MRA in the evaluation of CADs. Coronary MRA techniques, such as bright arterial lumen imaging and black arterial wall imaging, allow for high-resolution visualization of inflammatory vasculitis changes, including luminal stenosis, occlusion, mural thickening, and contrast enhancement of the affected vessel wall (151). Contrast enhancement of the coronary artery wall may indicate abnormalities of the vascular wall, including fibrosis, neovascularization, or higher permeability associated with inflammation (152).

While the clinical application of CMR for diagnosing CADs remains limited—reflected by only eight reports in the current literature—our preliminary study suggests its potential for visualizing and quantifying both subclinical and clinical coronary involvement. Notably, arterial wall imaging with coronary MRA can detect coronary wall enhancement before significant wall thickening develops, rendering it a valuable tool for early lesion identification. Beyond the morphological insights offered by CTA, coronary MRA provides additional diagnostic capabilities, helping to distinguish vascular inflammation from atherosclerosis based on wall-enhancement patterns. It may also provide a more precise assessment of the severity of coronary involvement and disease activity compared to CTA (128). In IgG4-RD patients, CMR not only enables the integrated identification of coronary involvement but also facilitates the evaluation of myocardial inflammatory infiltration or ischemic changes. In addition, CMR’s unique advantages in noninvasiveness and lack of ionization make it an ideal modality for longitudinal monitoring of disease progression, as well as evaluation of the effects of therapies. However, the clinical utility of CMR is currently constrained by its long scan times and the requirement for patient cooperation, which remain significant limitations for widespread application.

### 4.5 Adverse cardiovascular events caused by secondary stenosis

The findings reveal that the most common manifestation of coronary involvement in IgG4-RD is wall thickening and/or periarterial soft tissue formation. Despite the primary thickening in the coronary adventitia (153), nearly half of the patients in the cohort developed coronary narrowing (49%), which often led to myocardial ischemia or infarction. This narrowing subsequently led to clinical symptoms and was identified as the leading cause of cardiac death in the cohort. Notably, luminal stenosis was never observed in isolation, suggesting that the stenosis type (Type 4) is more likely a secondary manifestation of IgG4-RD coronary involvement. The development of stenosis in IgG4-RD appears to be partially related to mechanical compression from peri-coronary soft tissue formation due to infiltration of IgG4-positive plasma cells. This mechanism differs from other rheumatic diseases, including rheumatoid arthritis and lupus, where systemic inflammation accelerates atherosclerosis and subsequent coronary stenosis (59). However, it has also been proposed that IgG4-RD coronary arteritis and periarteritis may contribute to or accelerate atherosclerosis in affected arteries (84). In the cohort, 44% of cases were accompanied by atherosclerosis, with 20% of these cases leading to stenosis. Notably, among the atherosclerosis-associated cases, six patients had a history of CADs before their IgG4-RD diagnosis. This raises uncertainty regarding the extent to which the development of atherosclerosis in these patients can be attributed directly to IgG4-RD.

The critical role of the adventitia in vascular remodeling under pathological conditions has been well-documented. During inflammation, the adventitial region becomes a focal point for the local immune response, producing cytokines and enzymes that stimulate immune cell mobilization and accumulation (154). A cross-talk between intimal lesions and adventitial ATLOs via medial vascular smooth muscle cells has been suggested in atherosclerotic vessels (155). It can be reasonably hypothesized that this interaction between IgG4-related adventitial inflammation and the intima may contribute to the development of coronary atherosclerosis in IgG4-RD. Nevertheless, the precise mechanisms underlying stenosis in IgG4-RD coronary involvement remain complex and warrant further investigation. Advanced imaging techniques, including specific contrast agents combined with dedicated MRI pulse sequences, have shown promise in visualization of macrophage-rich inflammation within atherosclerotic plaques (156, 157). Additionally, coronary MRA has proven effective in visualizing and quantitatively assessing coronary artery lesions in IgG4-RD (128). Future studies integrating these advanced imaging modalities may provide valuable insights into the pathophysiological mechanisms of IgG4-RD coronary lesions and the connection between intimal lesions and adventitial inflammation.

It is also worth noting that, while the concurrent development of coronary aneurysms was less common, it was always accompanied by mural thrombosis, which could sometimes lead to thromboembolism and myocardial infarction. Interestingly, in two patients who died suddenly from myocardial infarction caused by thromboembolism, no aneurysms were observed, and only mild atherosclerosis was present, without definite localized luminal narrowing. It is speculated that the inflammatory state itself promotes the release of procoagulant substances, representing a critical risk factor for cardiovascular events. Therefore, it is necessary to carefully evaluate the coronary artery wall using CTA or CMR in IgG4-RD patients, particularly when DSA indicates no significant luminal stenosis.

### 4.6 The effect of treatments on IgG4-related coronary involvement and prognosis

Glucocorticoids remain the cornerstone of IgG4-RD treatment, demonstrating effectiveness in improving most cases of diffuse wall thickening and/or periarterial soft tissue encasement (158). Rituximab, by depleting B cells and reducing myofibroblast activation, offers the potential of alleviating the fibrotic processes associated with the disease (159). Studies have shown that rituximab can induce remission in 67–83% of cases and may facilitate early tapering of glucocorticoid therapy (160). For refractory or complex IgG4-RD cases, multiple immunosuppressants are currently available as adjunctive therapies to glucocorticoids. These immunosuppressants have been verified to be both safe and effective, particularly in patients resistant to corticosteroid treatment (161).

The prognosis of the aneurysm phenotype is often unsatisfactory and difficult to predict, representing the primary pattern of disease progression, which may be attributed to irreversible damage including coronary artery wall remodeling (84). While high-dose corticosteroids can suppress inflammation and hinder further development of IgG4-related arterial aneurysms, they do not reverse pre-existing and stable aneurysms lacking signs of active inflammation (162). Previous studies have suggested that high-dose corticosteroid treatment might cause arterial wall thinning, potentially raising the risk of aneurysm rupture (163). Immunosuppressive therapy is generally not recommended for coronary aneurysms, surgical intervention is typically prioritized, particularly in patients at high risk for rupture. However, performing revascularization surgery alone without concomitant lesion resection provides only symptomatic relief and carries a risk of progression, particularly in patients not maintained on subsequent corticosteroid therapy.

## 5 Limitations

Given the limited literature on IgG4-related coronary involvement, conducting a truly comprehensive systematic review remains inherently difficult. The study aimed to provide a detailed overview of the topic, particularly highlighting the role of imaging diagnostics, including CMR. However, the scarcity of available references on this specific subject poses a significant limitation. It is acknowledged that the limited number of studies, along with incomplete data on laboratory findings and patient prognoses in the existing literature, may have introduced bias into the conclusions. Nonetheless, this work may serve as a valuable foundation to stimulate further research. Studies with larger, more diverse, and well-characterized cohorts are essential for advancing understanding of this complex condition.

## 6 Conclusions

Coronary involvement of IgG4-RD could be categorized into four phenotypes, sometimes with an insidious onset and as the sole affected site, posing a potential risk for major adverse cardiovascular events. Integrating multimodal imaging is crucial for early diagnosis and effective monitoring, with coronary CMR showing promise for early detection without the risk of radiation-induced inflammation and fibrosis. Glucocorticoid therapy is effective for most patients, sometimes in combination with immunosuppressive therapy, while surgical intervention is prioritized for established aneurysms.

## Data Availability

The original contributions presented in the study are included in the article/**Supplementary Material**. Further inquiries can be directed to the corresponding authors.
