# Supplementary material for: Clinical characteristics and multimodal imaging insights of coronary involvement in immunoglobulin G4–related disease
Source: Front Immunol. 2025 Dec 4;16:1685508. doi: 10.3389/fimmu.2025.1685508 (PMC12711756; doi:10.3389/fimmu.2025.1685508)
Supplement: Supplementary file 1 [file DataSheet1.docx]

**Supplementary Material**

**Supplementary Tables**

**Table S1 Search strategy and results**

| **Databases** | **Date of search** | **Filters** | **Search strategy** | **Search results** |
| --- | --- | --- | --- | --- |
| Scopus | July 25, 2024 | English language, Medicine, Immunology and Microbiology, Exclude review and meta-analysis | (ALL (“Immunoglobulin G4–Related Diseases”) OR ALL (“IgG4 Related Systemic Disease”) OR ALL (“IgG4-RD”) OR ALL (“IgG4-Associated Autoimmune Disease”) OR ALL (“IgG4-Related Disease”)) AND (TITLE-ABS-KEY (“Coronary Vessel”) OR TITLE-ABS-KEY (“Coronary Arteries”) OR TITLE-ABS-KEY (“Coronary Artery”) OR TITLE-ABS-KEY (“Coronary Aneurysm”) OR TITLE-ABS-KEY (“Coronary periarteritis”)) | 230 |
| PubMed/MEDLINE | July 25, 2024 | Exclude review and meta-analysis, English language | (“Immunoglobulin G4–Related Diseases” [Title/Abstract] OR “IgG4 Related Systemic Disease” [Title/Abstract] OR “IgG4-RD” [Title/Abstract] OR “IgG4-Associated Autoimmune Disease” [Title/Abstract] OR “IgG4-Related Disease” [Title/Abstract]) AND (“Coronary Vessel” [Title/Abstract] OR “Coronary Arteries” [Title/Abstract] OR “Coronary Artery” [Title/Abstract] OR “Coronary Aneurysm” [Title/Abstract] OR “Coronary periarteritis” [Title/Abstract]) | 62 |
| Web of Science | July 25, 2024 | Exclude review and meta-analysis, English language | ((((TS= (Immunoglobulin G4–Related Diseases)) OR TS= (IgG4 Related Systemic Disease)) OR TS=(IgG4-RD)) OR TS= (IgG4-Associated Autoimmune Disease)) OR TS= (IgG4-Related Disease) AND ((((TS= (Coronary Vessel)) OR TS= (Coronary Arteries)) OR TS= (Coronary Artery)) OR TS= (Coronary Aneurysm)) OR TS= (Coronary periarteritis) | 185 |

**Table S2 Inclusion and exclusion criteria for study selection and screening**

| **Inclusion criteria** | **Exclusion criteria** |
| --- | --- |
| 1) Diagnoses should be definite following the classification criteria for 2019 American College of Rheumatology/European League Against Rheumatism. 2) Articles must contain description of coronary involvement in IgG4-RD, including crucial clinical data. 3) The articles should include imaging data of the patients’ coronary lesions. | 1) Systematic reviews or literature reviews. 2) No explicit evidence of coronary involvement or indirect coronary arteries abnormalities. 3) No specific pattern of coronary involvement. 4) Unable to get the essential data of each case. |

**Table S3 Other organ involvement excepting the cardiovascular system**

| Organ involvement | Number of patients |
| --- | --- |
| Lymph node | 36 |
| Pancreas | 21 |
| Lacrimal gland | 21 |
| Salivary gland | 20 |
| Kidney | 18 |
| Lung | 11 |
| Retroperitoneal | 9 |
| Biliary tract | 6 |
| Paranasal sinus | 2 |
| Prostate | 2 |

**Table S4 Characteristics of coronary lesion on CMR (eight published cases)**

| **Case (reference)** | **Age** | **Sex** | **Coronary lesions** |
| --- | --- | --- | --- |
| Lanzafame *et al*.^22^ | 63 | Male | Pseudo-mass surrounding LAD, isointense to myocardium in bSSFP cine-images, heterogeneously hypointense in T2-weighted sequences, and hypointense in T1-weighted sequences, with LGE. |
| Shibasaki *et al*.^27^ | 77 | Female | A substantial mass with cystic degeneration, surrounding RCA, spilling over into the diaphragm, enlarged to 50 × 55 mm outside the RV. |
| Liu *et al*.^40^ | 51 | Male | Abnormal mass surrounding the LAD, characterized by obscure boundaries and significant enhancement on LGE sequence. |
| Baruah *et al*.^49^ | 68 | Male | Heterogeneously enhancing mass lesion with enhanced perfusion and underlying aneurysmal dilatation of LAD. |
| Suwa *et al*.^88^ | 56 | Male | Double layers of the right coronary arterial wall were observed on gadolinium-enhanced PSIR images: high signal on the adventitial side and low signal on the luminal side. |
| Nishijo *et al*.^91^ | 46 | Male | Fat-suppressed gadolinium-enhanced T1-weighted imaging showed high gadolinium enhancement within the RCA aneurysm, T2-weighted imaging showed predominantly high signals with some low signals (thrombus). |
| Otani *et al*.^120^ | 83 | Male | Markedly enlarged outer layer thickness of the LAD and RCA was observed by cine-images and MR angiography. |
| Debonnaire *et al*.^113^ | 64 | Male | A giant LCX aneurysm and two smaller consecutive RCA aneurysms were identified by cine-images. |
| bSSFP: steady-state free precession; LGE: late gadolinium enhancement; RV: right ventricle; PSIR: phase sensitive inversion recovery. | | | |
